# Systematic-error suppression in low-coherence Brillouin optical correlation-domain reflectometry

**DOI:** 10.1038/s41598-023-44801-4

**Published:** 2023-10-16

**Authors:** Kenta Otsubo, Guangtao Zhu, Takaki Kiyozumi, Kohei Noda, Kentaro Nakamura, Heeyoung Lee, Yosuke Mizuno

**Affiliations:** 1 Faculty of Engineering, Yokohama National University, Yokohama, 240-8501 Japan; 2 Institute of Innovative Research, Tokyo Institute of Technology, Yokohama, 226-8503 Japan; 3 Graduate School of Engineering, The University of Tokyo, Tokyo, 113-8656 Japan; 4 College of Engineering, Shibaura Institute of Technology, Tokyo, 135-8548 Japan

**Keywords:** Imaging and sensing, Fibre optics and optical communications

## Abstract

Brillouin optical correlation-domain analysis (BOCDA) utilizing low-coherence light sources offers high-resolution distributed strain and temperature sensing. However, conventional BOCDA requires dual-end injection of pump and probe light into the sensing fiber. To overcome this limitation, low-coherence Brillouin optical correlation-domain reflectometry (BOCDR) based on spontaneous Brillouin scattering has emerged, enabling single-end light injection. While a pilot demonstration has shown a spatial resolution of 19 cm, a comparison of its measurement accuracy with standard BOCDR systems is yet to be explored. This study presents a distributed measurement with ~ 3 cm spatial resolution and demonstrates that low-coherence BOCDR eliminates systematic errors caused by direct sinusoidal modulation, offering enhanced measurement precision.

## Introduction

Brillouin scattering in optical fibers has been widely utilized for the development of distributed sensing systems due to the dependence of Brillouin frequency shift (BFS) on applied strain and ambient temperature^1^. Various spatially resolving techniques, including time-domain^2–5^, frequency-domain^6,7^, and correlation-domain^8–16^ methods, have been employed, each with its own advantages and limitations, as summarized in Refs.^17–19^. This study focuses on correlation-domain techniques, specifically Brillouin optical correlation-domain analysis (BOCDA)^9–11^ and Brillouin optical correlation-domain reflectometry (BOCDR)^12–16^, which offer high spatial resolution and random accessibility for strain and temperature measurements at arbitrary points along the sensing fiber.

BOCDA, requiring dual-end light injection into the sensing fiber, is based on stimulated Brillouin scattering (SBS), resulting in high signal-to-noise ratio (SNR) and spatial resolution. However, its setup is relatively complex, involving an electro-optic modulator (often using a single-sideband modulator to generate frequency-shifted probe light) and a lock-in amplifier. Moreover, if the sensing fiber breaks during operation, the measurement cannot be continued. In contrast, BOCDR only requires light injection from one end of the sensing fiber, relying on spontaneous Brillouin scattering. While BOCDR exhibits lower SNR and spatial resolution compared to BOCDA, its single-end accessibility enables convenient sensor embedding in structures with a relatively simple and cost-effective setup. Furthermore, in the event of sensing fiber breakage, BOCDR allows continuous measurement up to the point of breakage.

Efforts have been made in the past to enhance the spatial resolution of BOCDA by employing low-coherence light sources, where the coherence length determines the resolution. Although extremely high spatial resolution can be achieved, it comes at the cost of scanning the measurement position along the sensing fiber using a variable delay line, resulting in reduced operational speed. Notably, Hotate et al.^20^ demonstrated distributed strain measurement using low-coherence BOCDA with a noise-modulated laser, followed by Cohen et al.^21^, who utilized amplified spontaneous emission (ASE) from an erbium-doped fiber as a broader light source, achieving a resolution of 4 mm. Zafari et al.^22^ used ASE-based low-coherence BOCDA to measure BFS distribution in a chalcogenide waveguide with a resolution of 800 μm, while Wang et al.^23,24^ demonstrated low-coherence BOCDA using a chaotic laser with a resolution of 3.5 mm. Zadok et al.^25,26^ developed BOCDA with phase modulation by pseudo random bit sequence and achieved random-access distributed sensing without using a variable delay line. Despite the progress in achieving high spatial resolution, challenges such as two-end accessibility and high cost associated with probe light generation and lock-in detection in low-coherence BOCDA configurations still persist. Considering the relatively lower SNR of BOCDR, attaining such high resolutions may not be expected. Nevertheless, there is considerable interest in experimentally demonstrating the feasibility of low-coherence BOCDR with single-end accessibility.

To address this, Zhang et al.^27^ developed low-coherence BOCDR, referred to as noise-correlated Brillouin optical reflectometry in the literature. This system achieved distributed BFS measurement with a spatial resolution of 19 cm and an extended sensing range of ~ 250 m, a feature attributable to the specifications of the optical delay generator. However, due to the constraints of its minimal delay step size, the measurements were conducted at intervals of 30 cm along the sensing fiber. As a result, the realm of high-resolution distributed sensing has not been fully explored. While achieving higher spatial resolution is of some technical interest, it comes with the trade-off of a reduced SNR, offering insights into the potential capabilities of the low-coherence BOCDR. Moreover, although certain experimental parameters affecting the spatial resolution were investigated, the effect on the systematic errors caused by direct sinusoidal modulation of the light source, commonly observed in standard BOCDR systems^28–30^, was not assessed.

In this work, we utilize low-coherence BOCDR with a noise-modulated laser to perform distributed BFS measurements with an improved spatial resolution of approximately 3 cm. Through both simulation and experiment, we establish that the low-coherence BOCDR configuration is inherently resistant to the systematic errors caused by direct sinusoidal modulation of the laser. It is important to note that this paper complements the preliminary results presented in our previously submitted conference paper^31^. This work augments the existing literature by offering a comprehensive set of experimental results using pseudo-strain, simulation outcomes elucidating the mechanisms behind the suppression of systematic errors, and an in-depth analytical discussion.

## Principle and method

In standard BOCDR, the laser output undergoes sinusoidal frequency modulation using the synthesis of optical coherence functions technique^8^, generating correlation peaks along the sensing fiber^11^. Typically, the sensing fiber length is limited to maintain a single correlation peak, although exceptions have been reported^32,33^. By sweeping the modulation frequency, the position of the correlation peak can be scanned along the fiber, enabling distributed BFS measurements. The spatial resolution is determined by the modulation frequency and amplitude^12^.

In contrast, low-coherence BOCDR employs a low-coherence light source and generates a single correlation peak (0th order) at the point where there is no optical path length difference between the signal and reference paths. By altering the optical path length using a variable optical delay line, the sensing point can be scanned along the fiber for distributed measurements. The spatial resolution is dictated by the coherence length of the light source, as given by^34^

1 $$ \Delta z=\frac{2ln2}{\pi }\cdot \frac{c}{n\Delta f}, $$

where $\Delta z$ represents the spatial resolution, *c* is the speed of light in vacuum, *n* is the refractive index of the fiber core, and $\Delta f$ is the linewidth of the light source. Consequently, standard BOCDR and low-coherence BOCDR, both based on spontaneous Brillouin scattering, differ in the factors influencing spatial resolution and measurement range.

**Figure 1:**
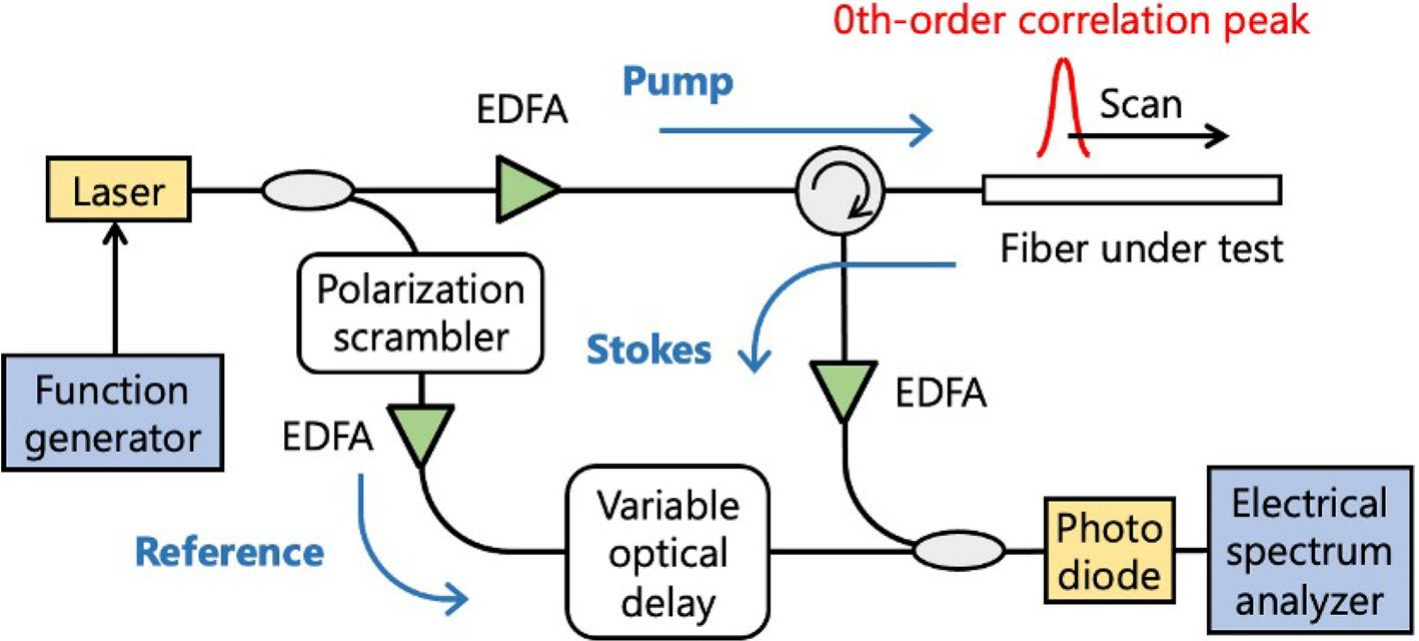
Experimental setup of low-coherence BOCDR. *EDFA* erbium-doped fiber amplifier.

Figure 1 illustrates the experimental setup of low-coherence BOCDR. Considering the lower signal-to-noise ratio of BOCDR compared to BOCDA, a noise-modulated distributed-feedback laser diode (LD) (WSLS-934010C1124-58, CivilLaser) with controllable linewidth through modulation amplitude was employed as the low-coherence light source, instead of using amplified spontaneous emission (ASE) or other broadband sources. The LD output was split into signal and reference light using an optical coupler. The signal light was amplified to 25.0 dBm by an erbium-doped fiber amplifier (EDFA) and injected into the sensing fiber through an optical circulator. The spontaneous Brillouin scattered light from the sensing fiber was further amplified to 1.8 dBm using another EDFA. The reference light underwent polarization scrambling, was amplified to 2.8 dBm, and passed through a variable optical delay line (75 cm in variable fiber length, ADL-200-25-SM-FS, Alnair Labs). Subsequently, it was mixed with the returned signal light for self-heterodyne detection. The resulting beat signal was converted to an electrical signal using a photodetector, and the Brillouin gain spectrum (BGS) was observed using an electrical spectrum analyzer (ESA). Distributed measurements were performed by continuously adjusting the length of the variable optical delay line to sweep the sensing position along the fiber.

## Experiments

**Figure 2:**
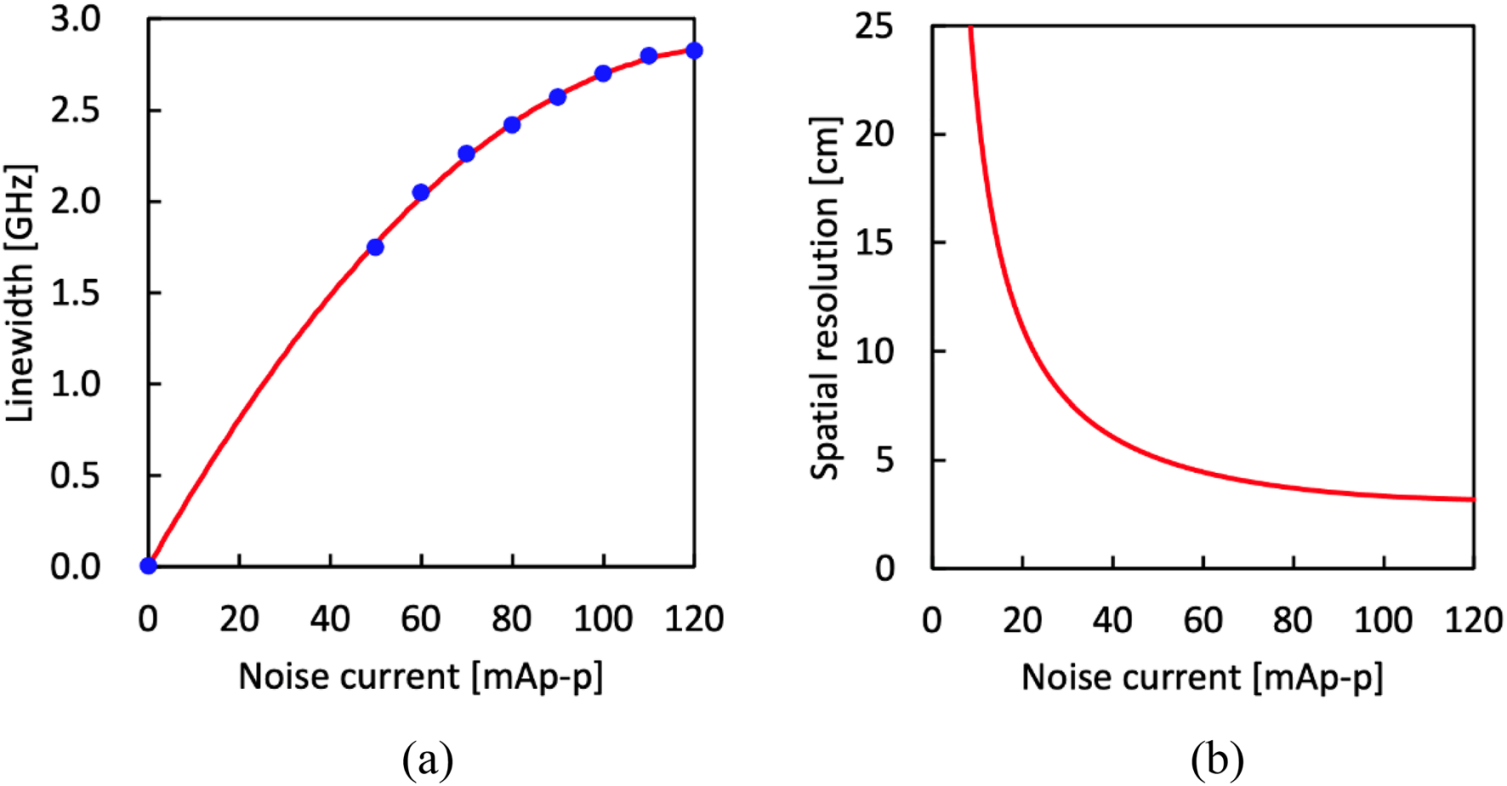
(**a**) Laser linewidth plotted as a function of the noise modulation current. The red solid curve is a polynomial fit. (**b**) Dependence of the theoretical spatial resolution on the noise modulation current.

Initially, the laser linewidth was measured as a function of the peak-to-peak current of the noise modulation, as depicted in Fig. 2a. The direct current bias was set to 140 mA. Due to the limited frequency resolution of the optical spectrum analyzer (AQ6370, Yokogawa Electric), the laser linewidth was directly measured only when the modulation current ranged from 50 to 120 mAp-p. The laser linewidth without modulation (~ 3 MHz, as specified in the datasheet) was also included in the plot. As the modulation current increased, the laser linewidth expanded, albeit with a decreasing dependence coefficient. These data points were fitted using a second-order polynomial curve. Figure 2b illustrates the theoretical spatial resolution calculated from the fitted curve and Eq. (1). As the modulation current increased, the theoretical spatial resolution improved gradually (with smaller values) and stabilized around 3 cm for higher modulation currents.

**Figure 3:**
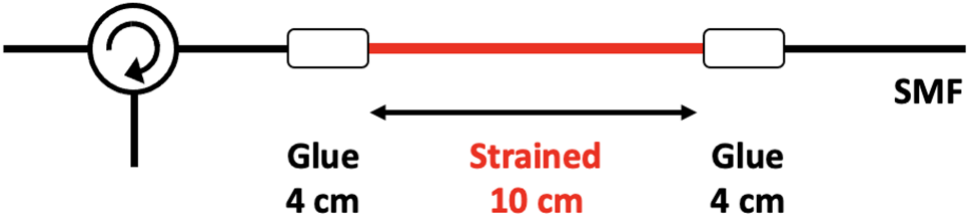
Structure of the sensing fiber used in the first measurement. *SMF* single-mode fiber.

Subsequently, two types of distributed strain measurements based on low-coherence BOCDR were demonstrated. In the first measurement, a sensing fiber with the structure shown in Fig. 3 was employed. Strains up to 1.0% were applied to a 10-cm-long section of a 4.4-m-long silica single-mode fiber (SMF) with a BFS of approximately 10.8 GHz at room temperature. The strained section was fixed on movable stages using two 4-cm-long glued parts, resulting in a total fixed length of 18 cm. The BGS and BFS distributions were measured along a 37.5-cm-long fiber segment, including the strained part, with 50 measurement points. The noise modulation current was set to 120 mAp-p, corresponding to a spatial resolution of 3.2 cm, as shown in Fig. 2b.

**Figure 4:**
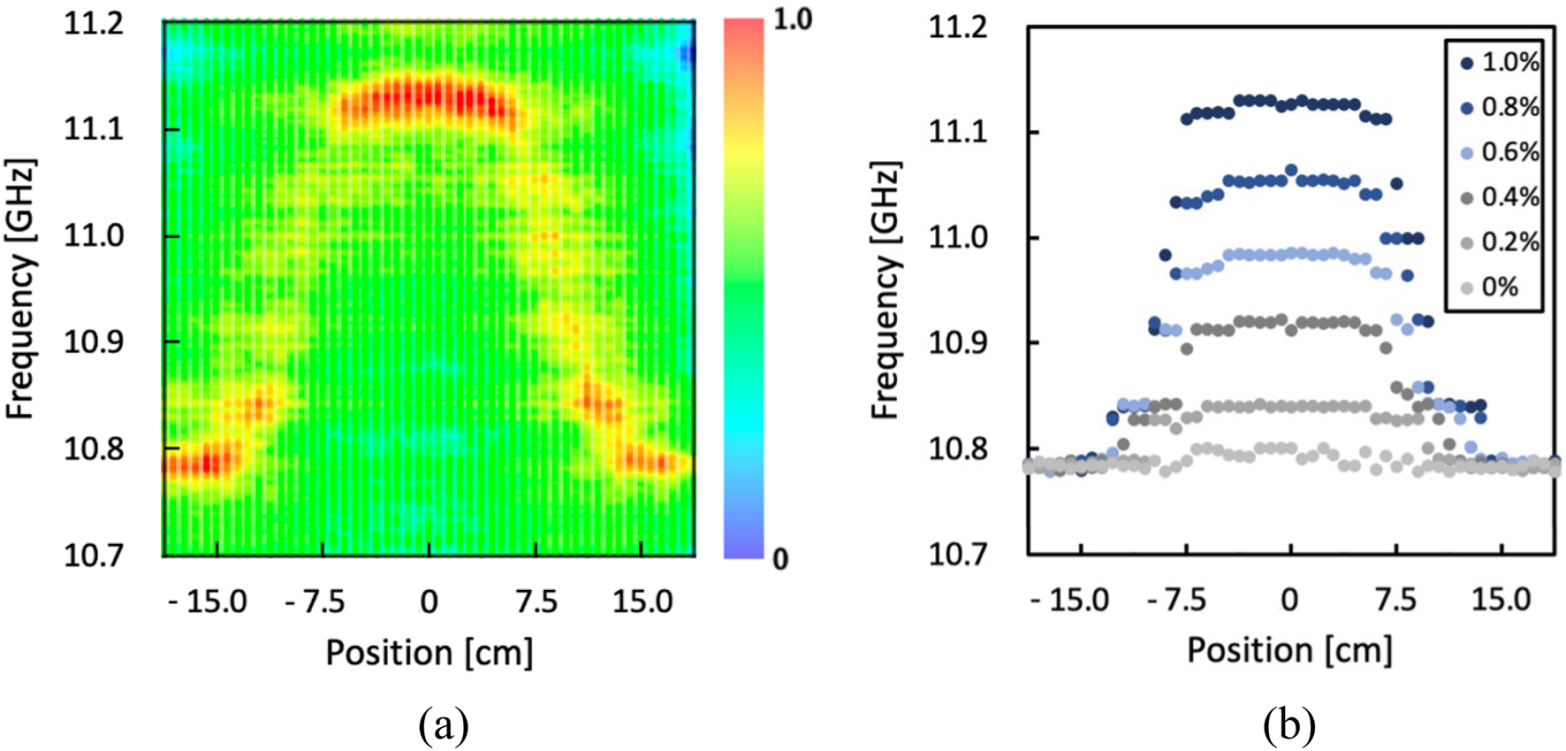
(**a**) Normalized BGS distribution measured when applied strain was 1.0%. (**b**) Measured BFS distributions with different applied strains.

Figure 4a, b present the normalized BGS distribution (measured at 1.0% strain) and the BFS distributions measured for different applied strains, respectively. Both figures clearly identify the strained region. With increasing applied strain, the BFS linearly shifted to higher frequencies, exhibiting a coefficient of 357 MHz/%, which is slightly lower than the typical value for silica SMFs. The gradual BFS shift reflects the strain distribution inside the glued parts, as indicated by the subsequent measurement. The lower strain coefficient of the BFS mentioned earlier is attributed to the dispersed strain rather than concentration solely on the strained section. The measured results exhibit high symmetry along the fiber length direction. In Fig. 4a, the skewness (absolute value) of the BFS distribution within this range is as low as 1.33 × 10^−5^. This observation indicates that the BFS distributions obtained through this technique are free from the distortion caused by the phase difference between amplitude modulation (AM) and frequency modulation (FM) often observed in standard BOCDR systems^28,29^.

**Figure 5:**
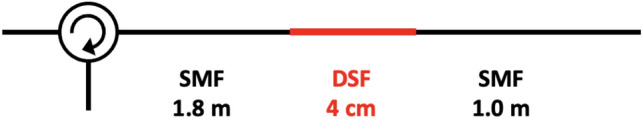
Structure of the sensing fiber used in the second measurement. *DSF* dispersion-shifted fiber.

In the second measurement, to eliminate the strain distribution induced in the glued parts and showcase higher spatial resolution, a sensing fiber with the structure depicted in Fig. 5 was utilized. A 4-cm-long dispersion-shifted fiber (DSF) with a BFS of approximately 10.5 GHz was spliced to 1.8 m- and 1.0-m-long SMFs to create a pseudo-strain. The noise modulation current was set to 120 mAp-p, corresponding to a theoretical spatial resolution of 3.2 cm. The BGS and BFS distributions were measured along a 10.0-cm-long fiber segment encompassing the DSF with 40 measurement points.

**Figure 6:**
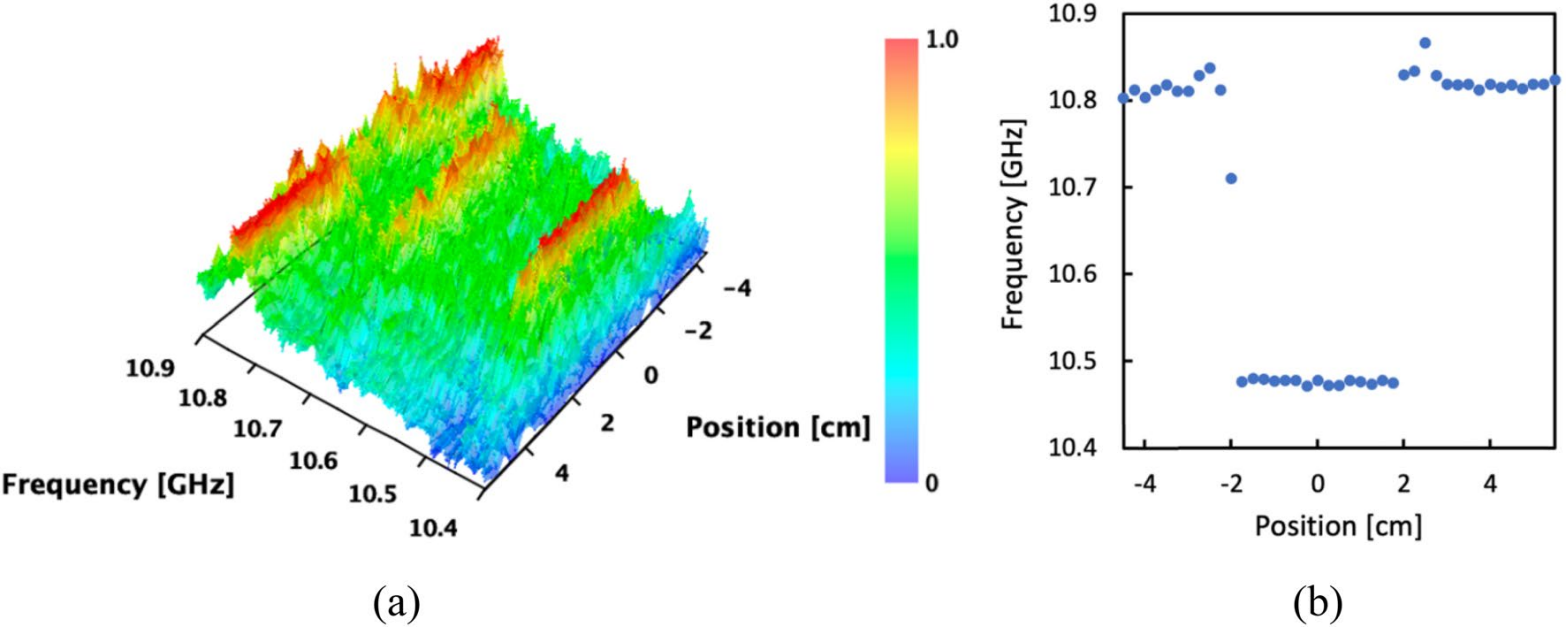
Measured distributions of (**a**) BGS (normalized) and (**b**) BFS.

The measured normalized BGS and BFS distributions are shown in Fig. 6a,b, respectively. Along a short section, the BFS shifted from approximately 10.81–10.48 GHz (matching the BFS of the DSF), and the length of the BFS-shifted section aligns well with the length of the DSF. At the boundaries between the SMF and DSF, the BFS experienced an abrupt change, which is distinct from the gradual change observed in Fig. 4 of the first measurement. It should be noted that the relatively prominent peak observed at approximately 10.65 GHz in the BGS corresponds to a higher-order Brillouin peak of the DSF. As its power consistently remains weaker than that of the main Brillouin peak, its influence on distributed measurements is negligible. Once again, the measured BFS distribution demonstrates a remarkable degree of symmetry along the fiber length direction, evident from the skewness (absolute value) of the BFS distribution in Fig. 6b, which measures 9.03 × 10^−4^. Our results indicate that the performance of this system remains unaffected by the AM-FM phase difference observed in standard BOCDR^28,29^. The high precision and reliability of distributed strain measurements achieved through low-coherence BOCDR are significant advantages of this technique.

### Simulation

To clarify the reason for the suppressed systematic errors in low-coherence BOCDR, we conducted a detailed analysis, following a method outlined in Ref.^30^, to calculate both the BGS and BFS distributions. We distinguish between “intrinsic BGS,” which represents the BGS at each position along the FUT, and “measured BGS,” which is the BGS observed using BOCDR. These two types of BGS distributions inherently differ. The measured BGS is derived from the square of the two-dimensional convolution of the “beat spectrum” and the intrinsic BGS distribution. The beat spectrum is defined as the power spectrum obtained from the Fourier transformation of the cross-correlation between the reference and Stokes light^13^.

**Figure 7:**
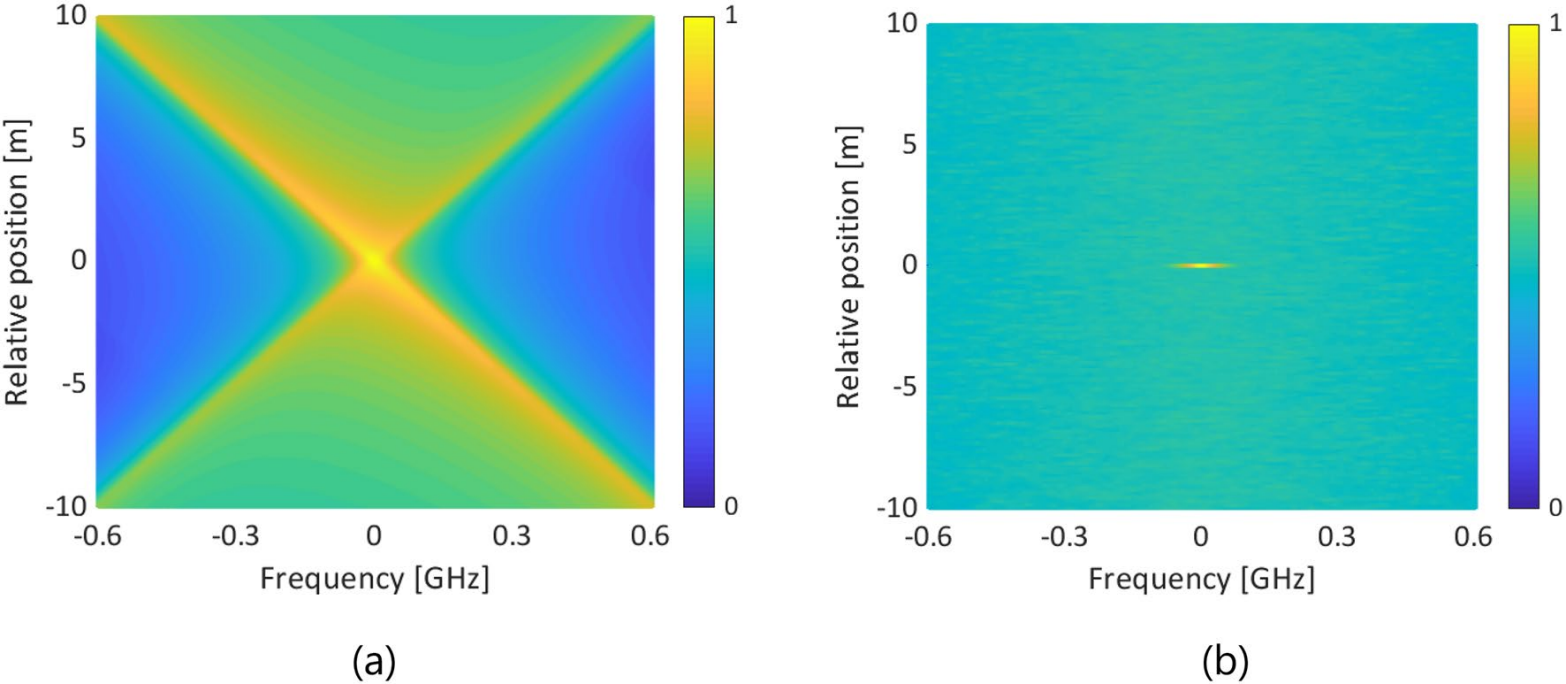
Comparative beat spectra for (**a**) standard BOCDR with sinusoidal modulation and (**b**) low-coherence BOCDR with noise modulation. The color bars show the normalized power.

The essential difference between standard BOCDR and low-coherence BOCDR lies in their respective beat spectra, generated by distinct modulation methods. A typical beat spectrum for standard BOCDR with sinusoidal modulation is depicted in Fig. 7a; the following conditions are assumed: FM amplitude = 1 GHz; modulation frequency = ~ 1 MHz; minimum-to-maximum AM ratio = 3 dB; AM-FM phase delay = π/4; nominal spatial resolution = ~ 1.0 m; measurement range = ~ 100 m; FUT length = 20 m. The beat spectrum exhibits a sharp correlation peak at the center, but some components spread in the four directions from the peak, and these components are not completely symmetrical because of the AM-FM phase delay. Conversely, a typical beat spectrum for low-coherence BOCDR with noise modulation is shown in Fig. 7b, where white noise with an amplitude of 1 GHz is assumed. It shows a similarly sharp correlation peak at the center, but with all the other components randomly scattered.

**Figure 8:**
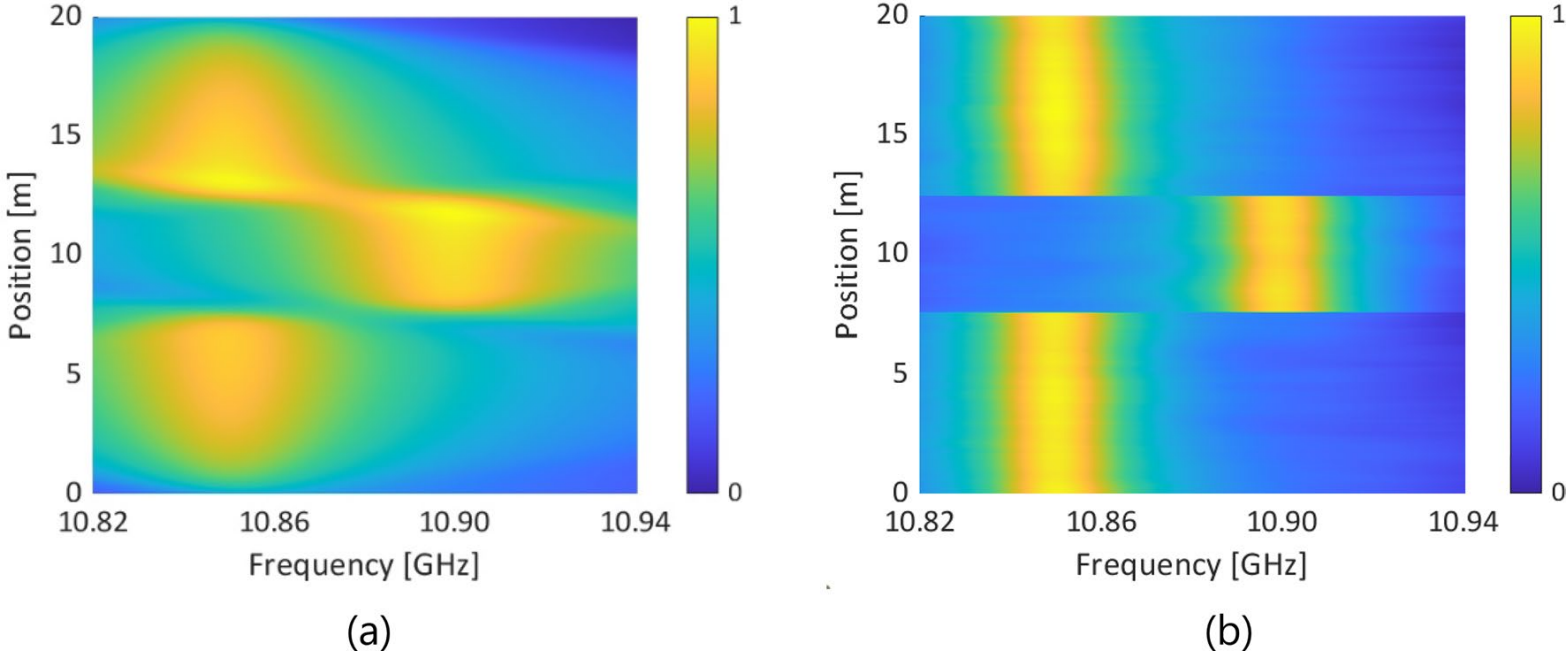
Measured BGS distributions of (**a**) standard BOCDR with sinusoidal modulation and (**b**) low-coherence BOCDR with noise modulation. The color bars show the normalized power.

**Figure 9:**
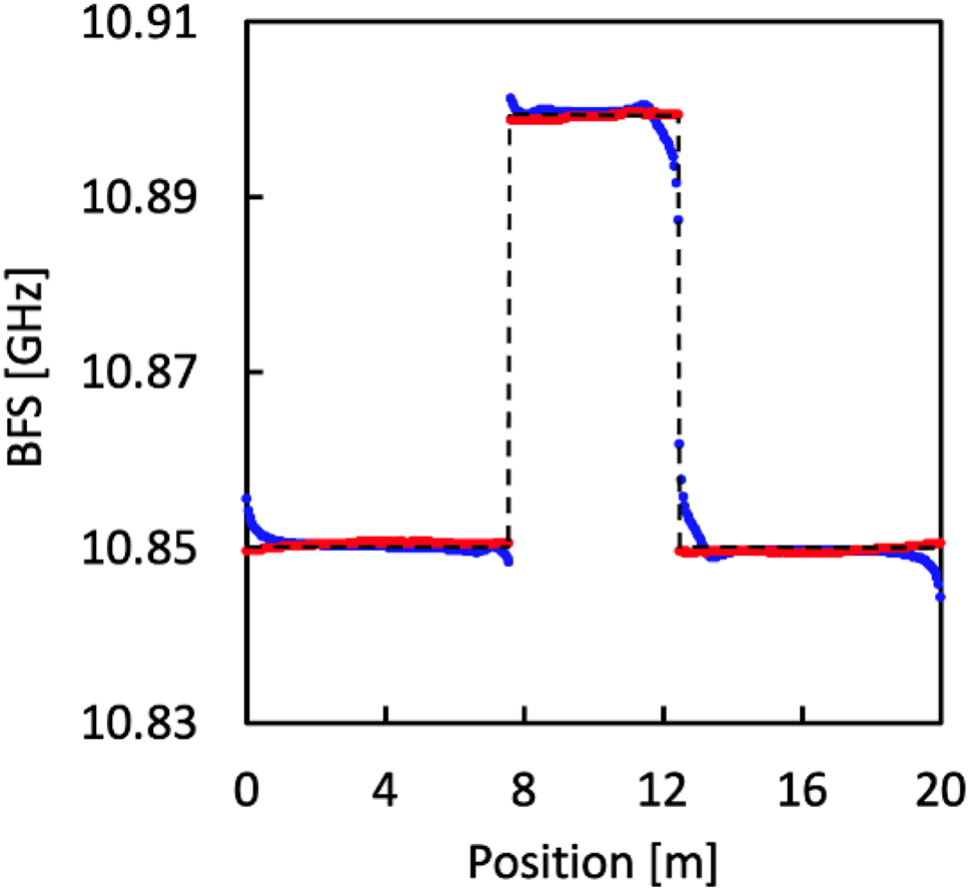
BFS distributions for standard BOCDR (represented in blue) and low-coherence BOCDR (represented in red). The black dotted lines indicate the intrinsic strain distribution, which closely aligns with the red plots.

By two-dimensional convolution of these beat spectra with the intrinsic BGS distributions (strained length = 4.9 m; strain = 0.11%, corresponding to a BFS change in a silica SMF of 50 MHz), we calculated the BGS and BFS distributions for both standard and low-coherence BOCDR. Our findings, illustrated in Fig. 8a,b (BGS distributions), and subsequently in Fig. 9 (BFS distributions), reveal that low-coherence BOCDR offers a more accurate BFS distribution, closely aligning with the intrinsic (or actual) strain distribution. Thus, our simulation elucidates the mechanisms by which low-coherence BOCDR mitigates the AM-FM phase delay-induced errors, primarily attributed to differences in beat spectra.

## Conclusion

We have demonstrated the effectiveness of low-coherence BOCDR utilizing a noise-modulated LD for distributed BFS measurements. With a spatial resolution of approximately 3 cm, our system surpasses the previous reported resolution by more than sixfold^27^. Through both simulation and empirical testing, we have substantiated that this approach effectively mitigates systematic errors arising from the AM-FM phase delay commonly encountered in direct sinusoidal laser modulation—a factor that often leads to distortions in BFS distributions in conventional BOCDR systems. While we acknowledge the current inconvenience associated with adjusting the variable delay line for sensing position control, we propose that this limitation could be overcome through the use of a periodically noise-modulated light source. Our research suggests that low-coherence BOCDR offers considerable promise for distributed strain measurements. Its multiple advantages—including single-end accessibility, enhanced spatial resolution, system simplicity, cost-effectiveness, and improved measurement accuracy—underscore its potential applicability and practicality across a broad range of use-cases.

## Data Availability

Data underlying the results presented in this paper are not publicly available at this time but may be obtained from authors upon reasonable request.
